# Epidemiological study of thyroid cancer at global, regional, and national levels from 1990 to 2021: an analysis derived from the Global Burden of Disease Study 2021

**DOI:** 10.3389/fendo.2025.1644270

**Published:** 2025-08-26

**Authors:** Lu Zhang, Liangliang Jiang, Rong Xu, Xuemei Zhang, Boxun Zhang, Rensong Yue

**Affiliations:** Hospital of Chengdu University of Traditional Chinese Medicine, Chengdu, Sichuan, China

**Keywords:** thyroid cancer, global disease burden, epidemiology, gbd2021, prediction

## Abstract

**Objective:**

A comprehensive evaluation of the disease burden is essential for identifying effective strategies to address thyroid cancer. This study delineates the long-term global trends in thyroid cancer and its epidemiological characteristics.

**Methods:**

Data on thyroid cancer from the Global Burden of Disease 2021 were utilized. The burden of thyroid cancer was assessed through measures of incidence, mortality, disability-adjusted life years (DALYs), and the socio-demographic index (SDI). Additionally, a global risk attribution analysis was conducted, and the Bayesian age-period-cohort (BAPC) model was employed to project the future global burden of thyroid cancer.

**Results:**

In 2021, there were an estimated 2 million (95% UI: 1.8, 2.2) cases of thyroid cancer worldwide, with an age-standardized prevalence rate of 23.1 (95% UI: 20.7, 25.6) per 100,000 individuals, reflecting a 55% increase since 1990. The global mortality from thyroid cancer in 2021 was 44,800 (95% UI: 39,900, 48,500), with an age-standardized rate of 0.5 per 100,000 people (95% UI: 0.5, 0.6), marking a 7% decrease since 1990. In the same year, the global total number of DALYs attributable to thyroid cancer was 1,246.5 thousand (95% UI: 1,094.4, 1,375.9), with an age-standardized rate of 14.6 per 100,000 population (95% UI: 12.8, 16.1), representing a 4.2% reduction compared to 1990.

**Conclusion:**

Over the past three decades, the age-standardized prevalence rate of thyroid cancer has increased, while the age-standardized mortality rate and DALY rate have decreased. Significant variations in prevalence, morbidity, and mortality exist across regions and countries. SDI plays a crucial role in the development of thyroid cancer, which is expected to remain a major public health challenge in the future.

## 1 Introduction

Thyroid carcinoma (TC) is a common endocrine malignancy, accounting for 3.4% of all newly diagnosed cancers annually (1). It ranks as the tenth most prevalent cancer worldwide (2). Thyroid cancer arises from the transformation of thyroid follicular cells into either differentiated or undifferentiated forms. Differentiated thyroid cancer constitutes approximately 90% of all malignant thyroid neoplasms, including follicular and papillary carcinomas (3). The pathogenesis of thyroid cancer is linked to the constitutive activation of key signaling pathways, including the mitogen-activated protein kinase (MAPK) and phosphatidylinositol-3-kinase/Akt (PI3K/AKT) pathways, which promote thyroid cell growth, proliferation, and survival (4). According to the European Society of Medical Oncology (ESMO) guidelines, the primary treatment for differentiated thyroid cancer involves thyroidectomy, lymph node dissection, and radioactive iodine therapy (5). Despite treatment, the recurrence rate of thyroid cancer remains substantial, with 25% to 30% of patients experiencing recurrence (6). As a result, thyroid cancer has become a major public health concern.

The Global Burden of Disease (GBD) project provides comprehensive epidemiological data that are invaluable for public health decision-making (7). The GBD database encompasses extensive information from 1990 to 2021, including prevalence, mortality, and disability-adjusted life years (DALY), which are essential for assessing global trends in thyroid cancer. This study aims to utilize GBD data to analyze the epidemiological patterns of thyroid cancer from 1990 to 2021 and to project future changes in its burden, thereby guiding public health policies and interventions.

## 2 Materials and methods

### 2.1 Sources of data

The GBD 2021 database provides updated estimates for 371 diseases and injuries, as well as 88 risk factors across 204 countries and territories worldwide (8). Data utilized in this study were obtained from the publicly accessible Global Health Data Exchange (GHDx) platform (https://vizhub.healthdata.org/gbd-results/). We extracted global data related to thyroid cancer, including information on age, incidence, mortality, and DALYs.

### 2.2 Statistical analysis and visualisation

To assess epidemiological trends in thyroid cancer, we analyzed data on prevalence, incidence, mortality, DALYs, and estimated annual percentage change (EAPC). Microsoft Excel 2024 was used to compile global thyroid cancer burden data from 1990 to 2021. Forecasting of future trends was performed using the Bayesian Age-Period-Cohort (BAPC) model. This model employs Bayesian inference to estimate long-term trends by incorporating the effects of age, period, and cohort. The BAPC model assumes that the age, period, and cohort effects are additive and independent, with no interactions between these dimensions. The core equation of the BAPC model is as follows:


$$log\left(Rate_{ijk}\right)=\alpha +\mu_{i}+\beta_{j}+\gamma_{k}+\epsilon_{ijt}$$

Rate_ijt_ represents the incidence or mortality rate at time t, for age group j, and cohort k. α is the intercept term. μ_i_ represents the period effect that varies over time i. β_j_ represents the age effect that varies across age group j. γ_k_ represents the cohort effect that varies across birth cohort k. ϵ_ijt_ is the error term, capturing unobserved variation or random fluctuations (9). Model validation was conducted using a hold-out approach, where the model’s predictive accuracy was assessed by comparing projected values with observed data excluded from model training, using mean absolute error (MAE) and root mean square error (RMSE) as evaluation metrics (10). The strength of the BAPC model lies in its capacity to accommodate data sparsity and heterogeneity, thereby producing more reliable predictive estimates.Model construction and inference were implemented using the BAPC package in RStudio, with parameters estimated through the Markov Chain Monte Carlo (MCMC) method. Additionally, the influence of the socio-demographic index (SDI) on thyroid cancer burden was evaluated. All data analyses and visualizations were performed using R version 4.4.1.

## 3 Results

### 3.1 International scale

In 2021, an estimated 2.0 million (95% uncertainty interval [UI]: 1.8–2.2) cases of thyroid cancer were reported globally, corresponding to an age-standardized prevalence rate of 23.1 per 100,000 population (95% UI: 20.7–25.6), representing a 55% increase compared to 1990. The global number of thyroid cancer-related deaths in 2021 was approximately 44,800 (95% UI: 39,900–48,500), with an age-standardized mortality rate of 0.5 per 100,000 population (95% UI: 0.5–0.6), indicating a 7% decrease since 1990. Additionally, thyroid cancer accounted for an estimated 1,246.5 thousand DALYs (95% UI: 1,094.4–1,375.9) in 2021, with an age-standardized DALY rate of 14.6 per 100,000 population (95% UI: 12.8–16.1), reflecting a 4.2% decline compared to 1990 (**Supplementary Table 1**).

### 3.2 Regional tier

In 2021, the age-standardized prevalence of thyroid cancer per 100,000 population was higher in High-income North America (45.5), Australasia (38.9), and the High-income Asia Pacific region (37.1) compared to Western Europe (32.7). In contrast, lower prevalence rates were observed in Western Sub-Saharan Africa (1.8), Central Sub-Saharan Africa (4.0), and Oceania (8.6) (**Supplementary Table 1**). During the same year, age-standardized mortality rates per 100,000 were relatively elevated in Andean Latin America (1.1), Eastern Sub-Saharan Africa (1.0), and Southeast Asia (0.9), whereas substantially lower rates were reported in Western Sub-Saharan Africa (0.1) and Central Sub-Saharan Africa (0.3) (**Supplementary Table 1**). Similarly, age-standardized DALY rates per 100,000 were highest in Eastern Sub-Saharan Africa (27.8), Andean Latin America (27.5), and Southeast Asia (23.6). In contrast, the lowest DALY rates were noted in Western Sub-Saharan Africa (2.8), East Asia (10.3), and Western Europe (10.5) (**Supplementary Table 1**).

Between 1990 and 2021, the most substantial increases in age-standardized prevalence of thyroid cancer were observed in Andean Latin America (188.1%) and South Asia (157.8%), while the most marked decline occurred in Central Europe (−6.9%) (**Supplementary Table 1**). Notable rises in age-standardized mortality rates were also recorded in South Asia (30.9%), Southern Sub-Saharan Africa (26.4%), and Andean Latin America (22.4%). In contrast, Central Europe experienced a considerable reduction (−49.1%), with further pronounced decreases observed in Western Europe (−42.8%) and Southern Latin America (−29.8%) (**Supplementary Table 1**). Similarly, the greatest increases in age-standardized DALY rates were seen in South Asia (28.2%), Southern Sub-Saharan Africa (26.3%), and Andean Latin America (19.6%). Conversely, significant declines in DALY rates were reported in Western Europe (−40.6%), Central Europe (−50.6%), and Southern Latin America (−29.6%) (**Supplementary Table 1**).

### 3.3 National level

**Figure 1:**
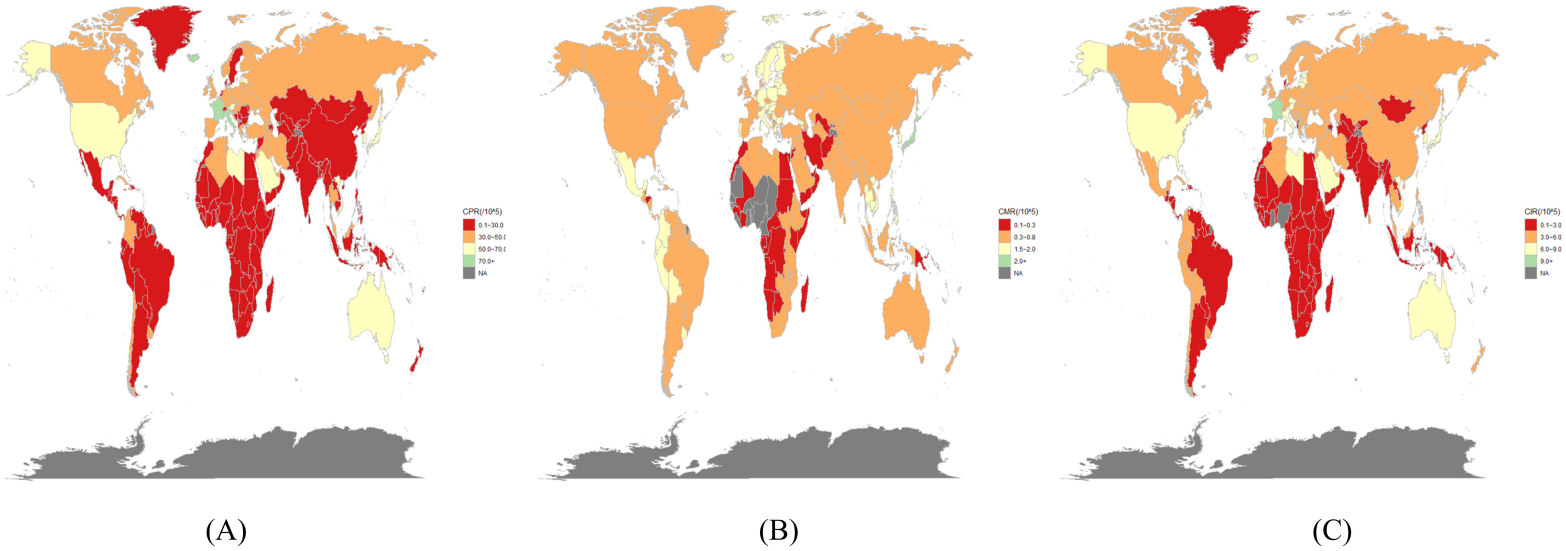
In 2021, the burden of thyroid cancer was estimated in 204 countries/territories. **(A)** prevalence, **(B)** mortality, and **(C)** incidence.

In 2021, the highest age-standardized prevalence of thyroid cancer per 100,000 population was reported in France (80.81 [95% UI: 66.71–96.48]), Italy (73.55 [95% UI: 66.42–81.28]), Iceland (71.70 [95% UI: 58.62–88.89]), and Taiwan (Province of China) (71.41 [95% UI: 61.34–81.32]) (**Figure 1A**). Countries with comparatively elevated age-standardized incidence rates included France (9.43 [95% UI: 7.79–11.24] per 100,000 population), Italy (8.88 [95% UI: 7.98–9.81]), and Iceland (8.73 [95% UI: 7.20–10.72]) (**Figure 1C**). Elevated age-standardized mortality rates were observed in Japan (1.80 [95% UI: 1.42–2.01] per 100,000 population), Latvia (1.48 [95% UI: 1.14–1.86]), and Georgia (1.47 [95% UI: 1.18–1.81]) (**Figure 1B**).

### 3.4 Demographic patterns of age and gender

**Figure 2:**
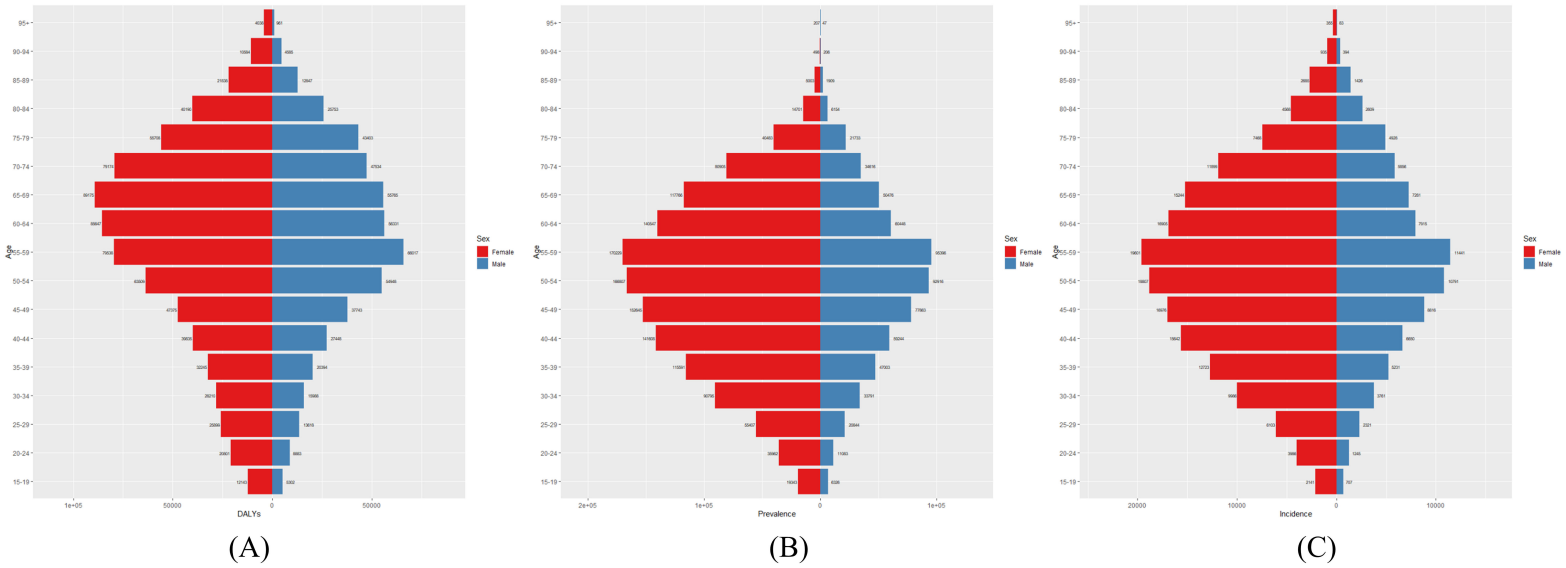
Global **(A)** Number of DALYs, **(B)** Number of Prevalences, and **(C)** Number of Incidences of thyroid cancer in 2021.

**Figure 3:**
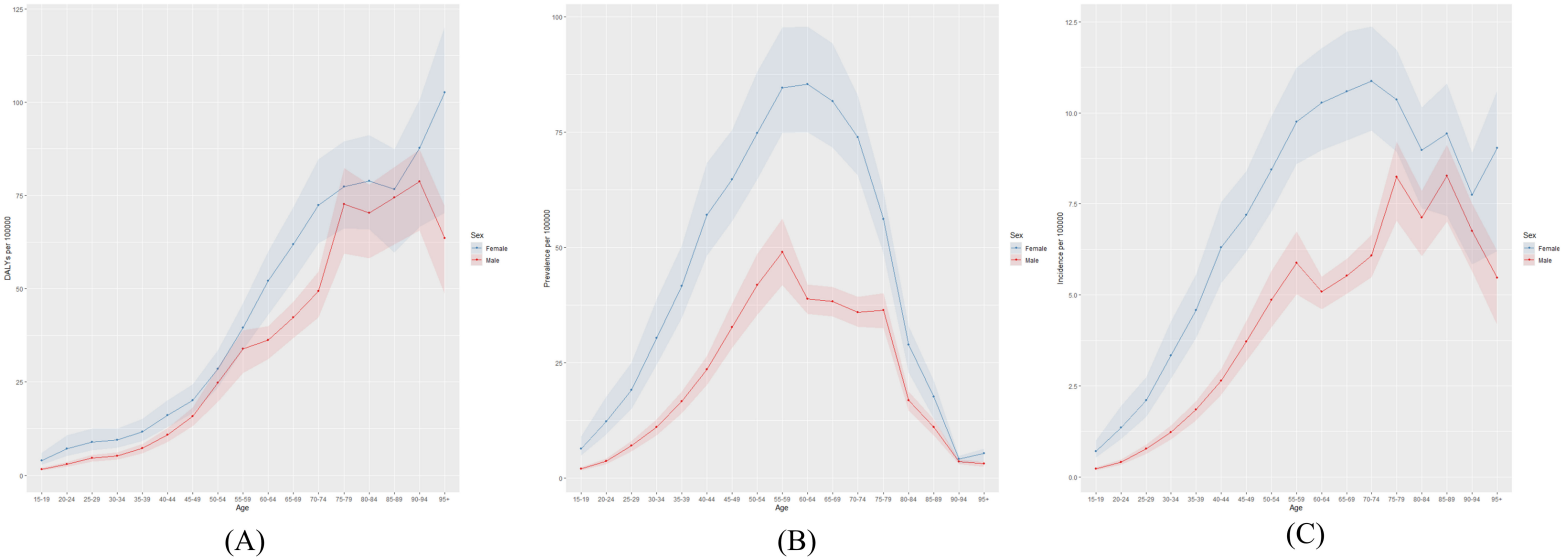
Global **(A)** age-standardized DALY rate, **(B)** age-standardized prevalence rate, and **(C)** age-standardized incidence rate of thyroid cancer in 2021.

**Figure 2** illustrates the global distribution of thyroid cancer burden in 2021, including the number of affected individuals, total cases, and associated DALYs. The highest number of DALYs was observed in women aged 65–69 years and in men aged 55–59 years. The largest number of cases occurred in individuals aged 55–59 years, regardless of sex. **Figure 3** presents the global age-specific prevalence, incidence, and DALY rates of thyroid cancer in 2021. DALY rates were higher in females than in males across most age groups. The highest prevalence was observed in women aged 60–64 years and in men aged 55–59 years.

### 3.5 Correlation with SDI

**Figure 4:**
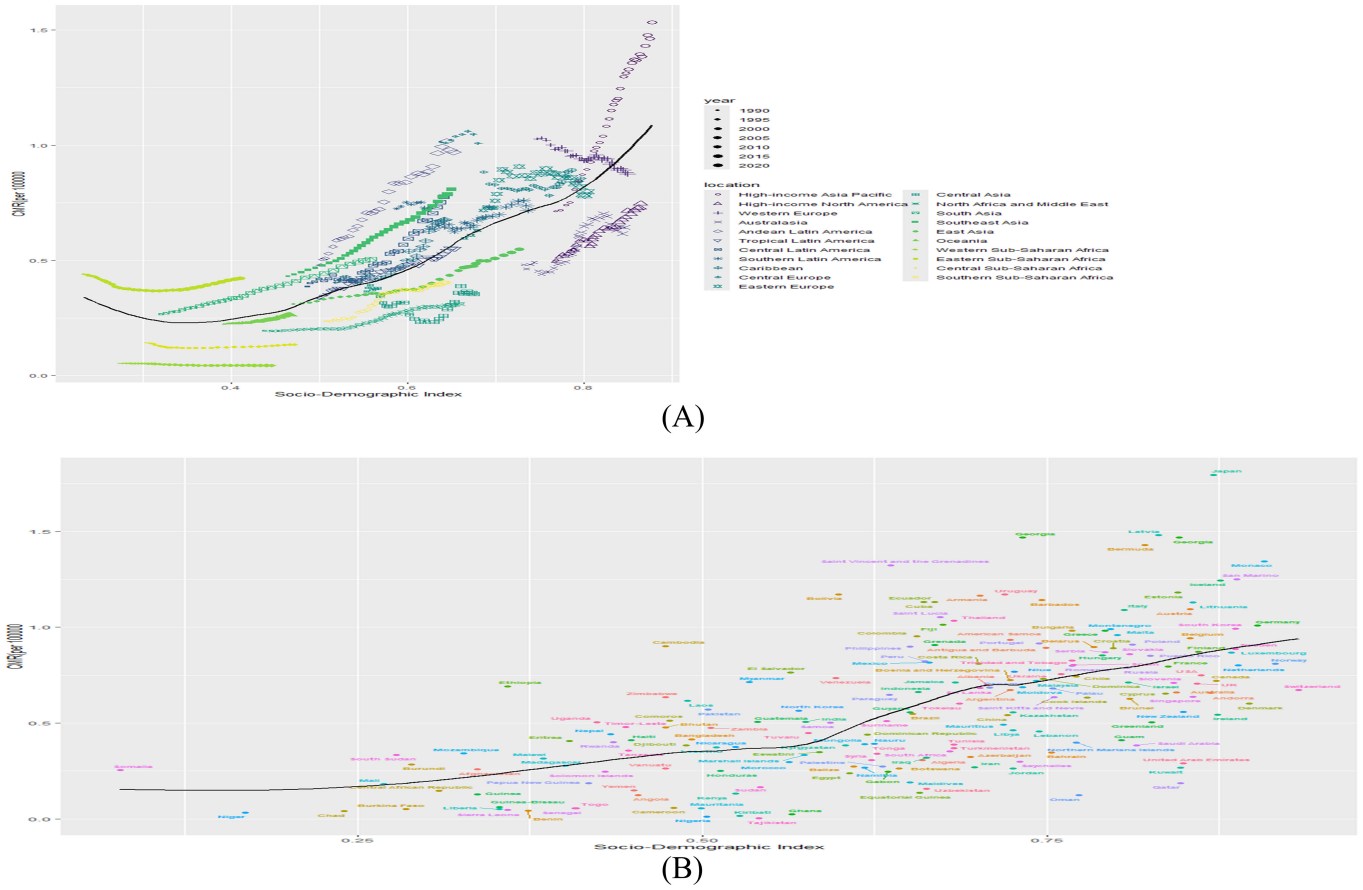
**(A)** Association of mortality with SDI by region, 1990-2021. **(B)** Association of prevalence with SDI by country in 2021. (B&L represents expected prevalence based on SDI only).

At the regional level, a U-shaped association was observed between the SDI and thyroid cancer mortality from 1990 to 2021, with mortality increasing markedly at both low and high levels of SDI. Notably, the observed prevalence exceeded expectations based on SDI in several regions, including Andean Latin America, High-income Asia Pacific, South Asia, Eastern Europe, and Southeast Asia (**Figure 4A**). At the national level, a V-shaped relationship was identified between thyroid cancer mortality and SDI. Countries such as Japan, Georgia, Latvia, and Italy demonstrated higher mortality rates than would be expected based on their respective SDI levels (**Figure 4B**).

### 3.6 Risk factors

**Figure 5:**
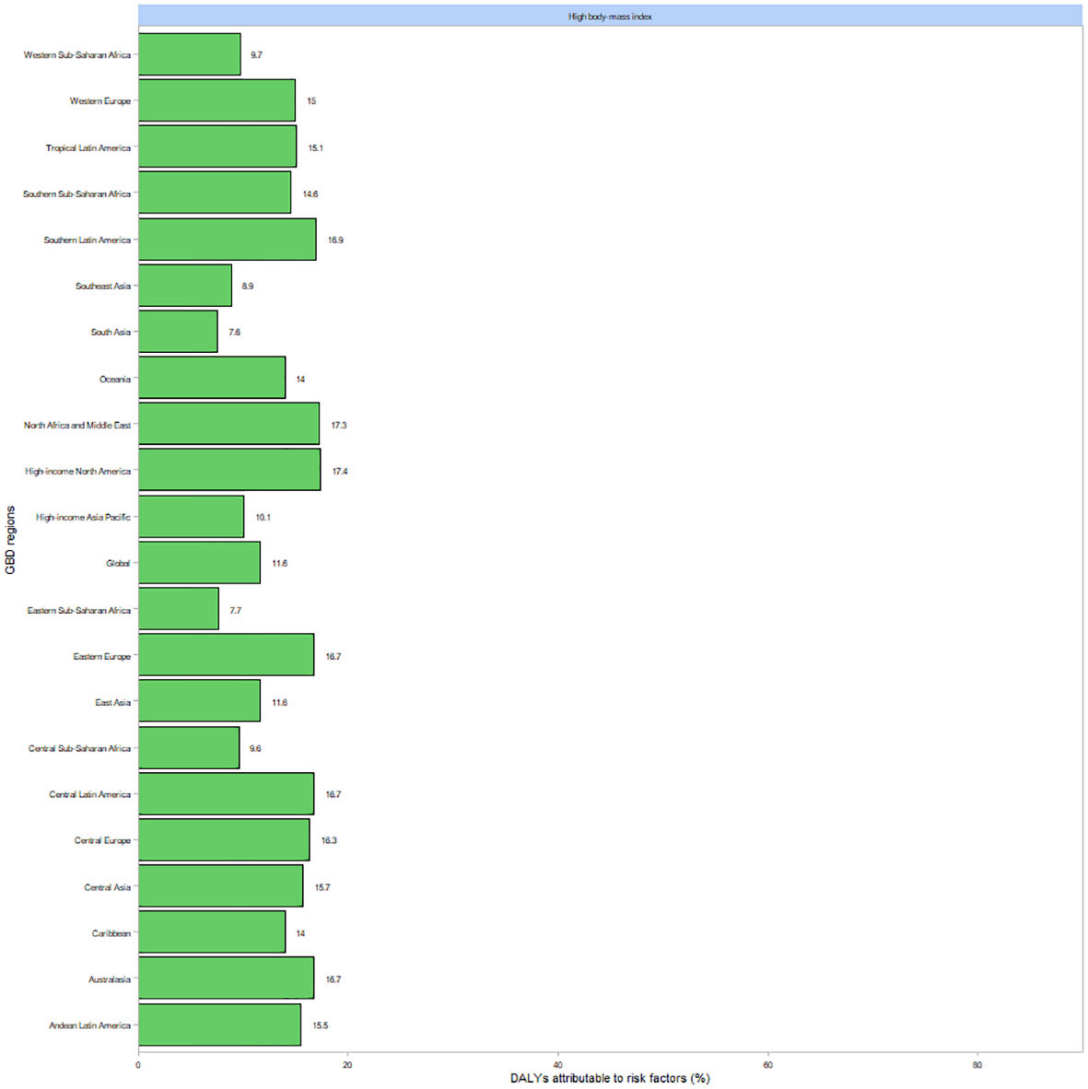
DALYs for thyroid cancer and risk factor shares in 21 regions in 2021.

**Figure 5** illustrates the proportion of DALYs attributable to risk factors for thyroid cancer globally and across 21 regions in 2021. Elevated body mass index (BMI) was the predominant contributing risk factor in Southern Latin America, North Africa and the Middle East, High-income North America, and Eastern Europe.

### 3.7 Prognostications

**Figure 6:**
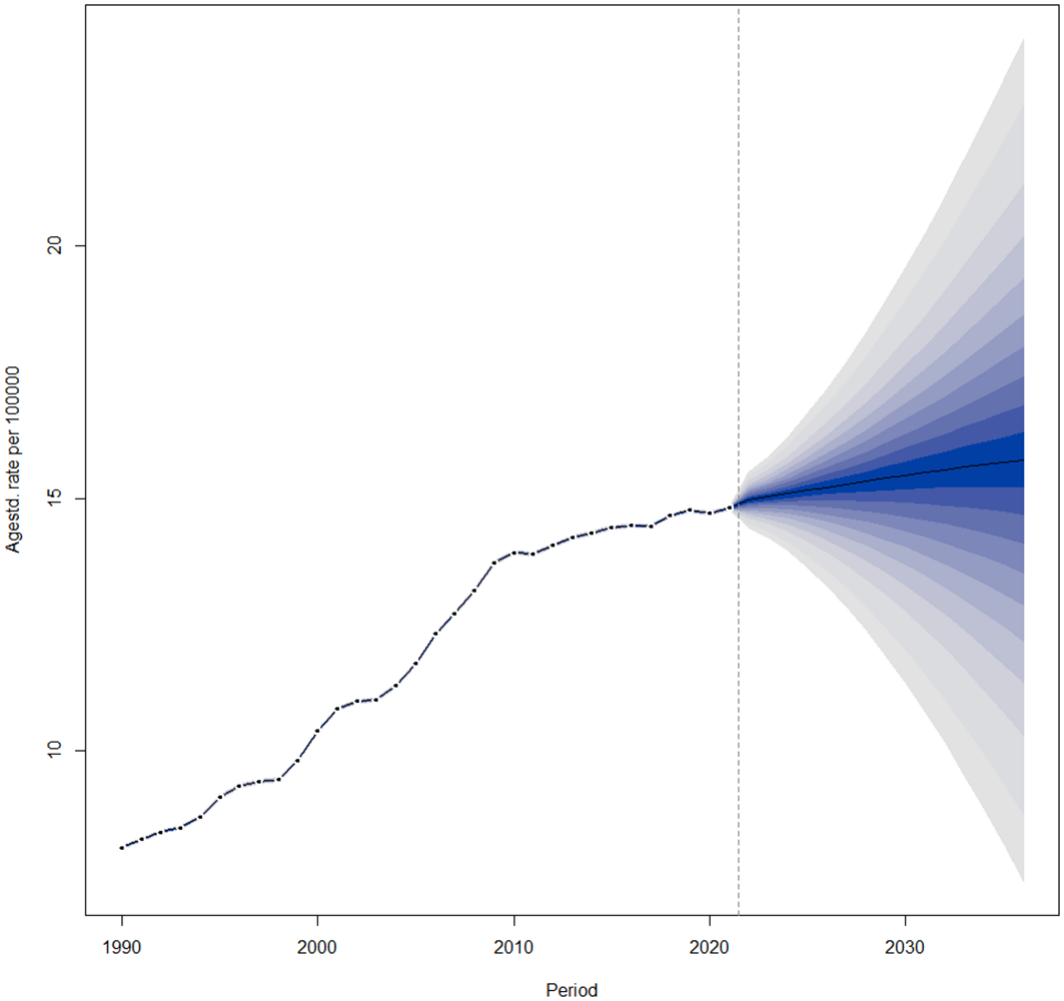
Temporal Trends in Global Age-Standardized Prevalence from 1990 to 2036.

The global age-standardized prevalence of thyroid cancer is projected to continue increasing from 2022 to 2036 (**Figure 6**).

## 4 Discussion

### 4.1 Principal discoveries

This report provides comprehensive statistics on the prevalence, mortality,DALYs, and age-standardized rates of thyroid cancer from 1990 to 2021, based on data from the Global Burden of Disease Study 2021. In 2021, an estimated 2 million cases, 44,800 deaths, and 1,246,500 DALYs were attributed to thyroid cancer worldwide. The burden of thyroid cancer was assessed at the global, regional, and national levels using contemporary epidemiological methods and risk factor analyses. These findings offer valuable insights into the impact of varying SDI levels on thyroid cancer burden and may inform policymakers in the development of targeted prevention and control strategies.

### 4.2 Comparison with alternative research

A 2024 report presented global estimates of the incidence, mortality, and DALYs associated with thyroid cancer from 1990 to 2021 (11). Building upon that work, we expanded the analysis by incorporating global prevalence data for the same period, examining key risk factors, and projecting future trends in disease burden. While several recent studies have compared the burden of thyroid cancer in China to that in the global context (12, 13), they did not comprehensively assess the global burden itself. In contrast, our study specifically focuses on the worldwide burden of thyroid cancer, offering a multifaceted evaluation that includes analysis by risk factors, SDI levels, and long-term forecasts. This study provides additional, timely, and policy-relevant insights to support global cancer control efforts.

Between 1990 and 2021, the global age-standardized prevalence of thyroid disorders has increased, with thyroid cancer emerging as a common malignancy. Thyroid cancer is classified into three major types: differentiated thyroid cancer, poorly differentiated and anaplastic thyroid cancer, and medullary thyroid cancer. Its etiology is primarily associated with genetic alterations, including gene amplification and copy number variations (4), somatic mutations (14), chromosomal rearrangements (15), aberrant DNA methylation (16), and dysregulation of non-coding RNAs (17). Standard treatment modalities for thyroid cancer include surgery, chemotherapy, radiation therapy, and radioiodine therapy (18). However, tumor recurrence and metastasis continue to pose major therapeutic challenges. The current treatment strategy increasingly incorporates targeted therapies, particularly combinations of tyrosine kinase inhibitors and immune checkpoint inhibitors (19). The global age-standardized mortality rate of thyroid cancer has slightly declined, which may be attributed to advancements in early diagnostic techniques for cervical lymph node metastasis. Early detection of cervical lymph node metastasis plays a critical role in the management of thyroid cancer, as it directly influences surgical indications and the extent of resection, as well as the risk of recurrence and overall survival (20). Several diagnostic techniques have enhanced the early detection of cervical lymph node metastasis, including the measurement of cytokeratin 19 fragment 21–1 in fine-needle aspiration (FNA) washout fluid (21), contrast-enhanced ultrasound (CEUS) (22), sentinel lymph node biopsy (SLNB) (23), and artificial intelligence-based approaches such as deep learning (24). These methods have improved the accuracy of preoperative assessment and reduced thyroid cancer-related mortality.

At the regional level, high-income North America exhibits the highest prevalence of thyroid cancer. Overdiagnosis of thyroid cancer is particularly common in this region (25). The widespread use of ultrasound and FNA biopsy, along with advanced imaging modalities such as computed tomography (CT) and magnetic resonance imaging (MRI), has contributed to increased detection of thyroid nodules and early-stage thyroid cancers (26). In high-income North America, medical radiation exposure is notably elevated, and exposure to ionizing radiation in the head and neck region is a well-established risk factor for thyroid cancer (27). Additionally, individuals of Caucasian descent in this region are believed to carry genetic predispositions that significantly increase their susceptibility to thyroid cancer (28). Obesity, which is highly prevalent in high-income North America, is another important risk factor associated with increased thyroid cancer incidence (29, 30).

In 2021, France reported the highest prevalence of thyroid cancer. It has become the sixth most commonly diagnosed malignancy among women in the country (31). Reports indicate that approximately 78% of French patients with thyroid cancer undergo total thyroidectomy as the primary treatment, and the associated medical expenses are higher for male patients than for female patients. Overdiagnosis is estimated to occur in 71.2% of individuals diagnosed with thyroid carcinoma in France (32). Li et al. investigated the global impact of thyroid cancer overdiagnosis, estimating that it affects approximately 1.7 million individuals worldwide (33). In France, the increased use of diagnostic modalities such as thyroid ultrasonography, radionuclide scanning, and cytological evaluation has contributed to the rising detection rate of thyroid cancer (34). Statistically, the estimated overdiagnosis rate among French men and women ranges from 70% to 80%, underscoring the significant influence of diagnostic advancements on the observed incidence (35).

Emerging evidence suggests that a high BMI is a significant risk factor for thyroid cancer. Emily Peterson et al. (36) investigated the relationship between BMI and thyroid cancer and identified a positive association, with risk estimates ranging from 1.1 to 2.3 in men and from 1.0 to 7.4 in women. One proposed mechanism is that insulin and insulin-like growth factor I (IGF-I) stimulate thyroid cell proliferation, thereby contributing to carcinogenesis (37). In addition, elevated thyroid-stimulating hormone (TSH) levels observed in obese individuals have been found to play a substantial role in the development of thyroid cancer (38). Excess adipose tissue may promote cancer cell proliferation and metastasis through mechanisms involving insulin resistance and IGF-I release, and it may exert a direct stimulatory effect on thyroid cell growth (39). Furthermore, the dietary patterns of individuals with obesity—often characterized by high protein and carbohydrate intake—have been associated with increased thyroid cancer risk (40). A strong correlation between the prevalence of thyroid cancer and the SDI has also been established. At both regional and national levels, thyroid cancer prevalence has been observed to increase with rising SDI levels. In economically developed regions and countries equipped with advanced medical technology, increased exposure to medical radiation during diagnostic procedures is believed to contribute to the rising incidence of thyroid cancer.Moreover, thyroid cancer mortality increases significantly with higher SDI, which may reflect a shift toward more aggressive tumor behavior. Higher SDI regions also exhibit a greater prevalence of obesity, a condition that is positively associated with both thyroid cancer risk and aggressiveness (41). Obesity has been shown to promote more aggressive forms of thyroid cancer (42, 43), potentially through altered thyroid function. In particular, TSH levels are often elevated in obese individuals, and elevated TSH has been linked to increased thyroid cancer aggressiveness (44).

The strength of this study lies in its systematic and comprehensive evaluation of the epidemiology of thyroid cancer at global, regional, and national levels from 1990 to 2021. It provides a comparative assessment of thyroid cancer prevalence across countries and regions with varying levels of medical resources. Regions and countries with a high burden of thyroid cancer should strengthen their healthcare infrastructure and adapt health policies to improve the prevention and management of the disease.However, this study has several limitations. The analysis is based on data obtained from the GBD database, and data quality is subject to variability in surveillance capacity across countries and regions. Moreover, the GBD database does not provide histological stratification of thyroid cancer, which limits the granularity of our findings. Additionally, inconsistencies in data quality and limited availability of raw data from certain countries may affect the accuracy and reliability of the results.

## 5 Conclusion

This study investigated the global epidemiological patterns and trends of thyroid cancer over the past three decades. While the age-standardized prevalence rate demonstrated an upward trajectory, both the age-standardized mortality rate and the age-standardized DALY rate exhibited a declining trend. Marked disparities in prevalence, morbidity, and mortality were observed across different regions and countries. The SDI was found to significantly influence thyroid cancer prevalence, indicating that thyroid cancer will remain a major public health concern in the foreseeable future.

## Data Availability

Publicly available datasets were analyzed in this study. This data can be found here: The data for this study are available in the GBD database (https://vizhub.healthdata.org/gbd-results/).
