# Supplementary material for: Epidemiological study of thyroid cancer at global, regional, and national levels from 1990 to 2021: an analysis derived from the Global Burden of Disease Study 2021
Source: Front Endocrinol (Lausanne). 2025 Aug 26;16:1644270. doi: 10.3389/fendo.2025.1644270 (PMC12417126; doi:10.3389/fendo.2025.1644270)
Supplement: Supplementary file 1 [file Table1.docx]

Table 1 The prevalence, mortality, and disability-adjusted life years (DALYs) of thyroid cancer in 2021, as well as the percentage change in age-standardized rates (ASR) per 100,000 people in global disease burden regions from 1990 to 2021.

|  |  | Prevalence (95% UI) |  |  | Deaths (95% UI) |  |  | DALYs (95% UI) |  |  |
| --- | --- | --- | --- | --- | --- | --- | --- | --- | --- | --- |
|  | Location | No, in millions (95% UI) | ASRs per 100 000 (95% UI) | Percentage change in ASRs from 1990 to 2021 | No, in thousands (95% UI) | ASRs per 100 000 (95% UI) | Percentage change in ASRs from 1990 to 2021 | No, in thousands (95% UI) | ASRs per 100 000 (95% UI) | Percentage change in ASRs from 1990 to 2021 |
| 1 | Global | 2 (1.8,2.2) | 23.1 (20.7,25.6) | 55 (41.3,69.9) | 44.8 (39.9,48.5) | 0.5 (0.5,0.6) | -7 (-15.8,0.1) | 1246.5 (1094.4,1375.9) | 14.6 (12.8,16.1) | -4.2 (-14.1,5.5) |
| 2 | High-income Asia Pacific | 0.1 (0.1,0.1) | 37.1 (33.2,43.8) | 37.8 (23.3,57.8) | 2.8 (2.3,3.2) | 0.5 (0.4,0.6) | -21.6 (-29.6,-15.1) | 50.8 (43.7,57.6) | 11.8 (10.5,13.7) | -23.1 (-29.9,-15.6) |
| 3 | High-income North America | 0.2 (0.2,0.2) | 45.5 (43.6,47.3) | 41.9 (35.8,48.5) | 2.8 (2.5,2.9) | 0.4 (0.4,0.4) | 5.5 (1.3,9.8) | 70.6 (65,76.5) | 12 (11,13) | 6.5 (2.2,10.9) |
| 4 | Western Europe | 0.2 (0.2,0.2) | 32.7 (29.9,35.5) | 5.2 (-4.7,15.5) | 3.8 (3.3,4.2) | 0.4 (0.3,0.4) | -42.8 (-47.2,-38.1) | 84.5 (75.8,92.6) | 10.5 (9.4,11.5) | -40.6 (-45.3,-35.4) |
| 5 | Australasia | 0 (0,0) | 38.9 (31.4,47.6) | 88.7 (48,134.9) | 0.2 (0.2,0.2) | 0.4 (0.3,0.4) | -12.8 (-29,7.2) | 5 (4.1,6) | 10.6 (8.7,12.8) | -3.9 (-22.3,17.5) |
| 6 | Andean Latin America | 0 (0,0) | 28.1 (21.6,35.6) | 188.1 (119.9,282.2) | 0.6 (0.5,0.8) | 1.1 (0.9,1.4) | 22.4 (-2.1,54.5) | 16.7 (13.2,20.8) | 27.5 (21.7,34.3) | 19.6 (-5,53) |
| 7 | Tropical Latin America | 0 (0,0) | 12.6 (11.8,13.3) | 61.6 (50.2,72.8) | 1.3 (1.1,1.3) | 0.5 (0.5,0.5) | -14.3 (-20.1,-9.2) | 32.7 (30.5,34.7) | 12.7 (11.8,13.5) | -14.2 (-19.5,-8.5) |
| 8 | Central Latin America | 0.1 (0.1,0.1) | 21.8 (19.4,24.6) | 113.5 (87.8,140.4) | 2 (1.7,2.2) | 0.8 (0.7,0.9) | 1.4 (-9.1,12.4) | 52.2 (47,58.1) | 20.4 (18.4,22.7) | 4.4 (-6.8,15.9) |
| 9 | Southern Latin America | 0 (0,0) | 19.7 (17.1,22.6) | 39.1 (20,62.4) | 0.5 (0.4,0.6) | 0.6 (0.5,0.6) | -29.8 (-39.1,-19.1) | 12 (10.5,13.6) | 14.3 (12.5,16.2) | -29.6 (-38.7,-18.5) |
| 10 | Caribbean | 0 (0,0) | 17.8 (15.4,20.6) | 67.4 (41.5,95.4) | 0.3 (0.3,0.4) | 0.6 (0.5,0.7) | 8.2 (-5.2,23.3) | 8.7 (7.5,10.1) | 16.3 (14.1,19) | 9.6 (-5.3,26.3) |
| 11 | Central Europe | 0 (0,0) | 21.9 (19.7,24) | -6.9 (-16.3,3.1) | 1 (0.9,1.1) | 0.4 (0.4,0.5) | -49.1 (-53.3,-44.7) | 23.4 (21.3,25.5) | 11.6 (10.5,12.7) | -50.6 (-55,-46) |
| 12 | Eastern Europe | 0.1 (0.1,0.1) | 25.9 (23.3,28.8) | 37 (22.1,52.8) | 1.7 (1.5,1.8) | 0.5 (0.4,0.5) | -4.8 (-14.2,4.4) | 42.1 (38.2,46.4) | 12.9 (11.7,14.1) | -7.1 (-16.7,1.9) |
| 13 | Central Asia | 0 (0,0) | 13.4 (11.7,15.2) | 5.3 (-10.3,22.9) | 0.3 (0.3,0.4) | 0.4 (0.4,0.5) | -19.7 (-29.3,-8.2) | 10.3 (9.1,11.5) | 11.7 (10.3,13) | -26.5 (-35.8,-15.7) |
| 14 | North Africa and Middle East | 0.2 (0.2,0.2) | 30.7 (25.4,36) | 135.3 (78.2,199.2) | 1.9 (1.7,2.2) | 0.4 (0.4,0.5) | 10.2 (-17,36) | 65.3 (55.5,76.5) | 12.7 (10.9,14.8) | 15.4 (-14.4,44.3) |
| 15 | South Asia | 0.3 (0.2,0.3) | 15.3 (12.3,18.5) | 157.8 (95.9,226) | 9.3 (7.8,10.7) | 0.6 (0.5,0.7) | 30.9 (3.7,55.9) | 302.3 (249.8,356.8) | 18.2 (15.1,21.4) | 28.2 (0.9,56) |
| 16 | Southeast Asia | 0.2 (0.2,0.2) | 26.9 (21.1,31.8) | 111.3 (77.8,145.6) | 5.6 (4.6,6.5) | 0.9 (0.7,1) | 11.1 (-4.4,27.4) | 164.5 (130.3,189.2) | 23.6 (18.8,27) | 10.3 (-5,25.9) |
| 17 | East Asia | 0.4 (0.3,0.5) | 20.5 (16.8,25.6) | 139.9 (86.4,225) | 8.1 (6.5,9.8) | 0.4 (0.3,0.5) | -18.1 (-33.6,3.3) | 213.6 (173.1,262.4) | 10.3 (8.3,12.5) | -16.3 (-32.8,7.5) |
| 18 | Oceania | 0 (0,0) | 8.6 (5.2,12.3) | 26.1 (0,57.3) | 0 (0,0.1) | 0.5 (0.4,0.7) | -3.7 (-21.9,18.1) | 1.2 (0.7,1.6) | 13.5 (8.6,18.6) | -2.6 (-20.8,19.4) |
| 19 | Western Sub-Saharan Africa | 0 (0,0) | 1.8 (1.3,2.3) | 42 (8.5,79.1) | 0.2 (0.2,0.3) | 0.1 (0.1,0.1) | -11.6 (-29.2,12.3) | 8 (6.1,10.3) | 2.8 (2.2,3.5) | -11.6 (-30.3,11.9) |
| 20 | Eastern Sub-Saharan Africa | 0 (0,0.1) | 15.4 (11.2,23.1) | 64.3 (19.8,143.2) | 1.8 (1.3,2.5) | 1 (0.7,1.3) | -1.7 (-24.3,29.3) | 66.5 (48.5,94.8) | 27.8 (20.6,38.4) | -7.6 (-30.6,26) |
| 21 | Central Sub-Saharan Africa | 0 (0,0) | 4 (2.5,6.3) | 52.3 (10.4,111.3) | 0.2 (0.1,0.3) | 0.3 (0.2,0.6) | -3.3 (-28.4,27.2) | 6 (3.9,9.2) | 8.9 (5.8,13.9) | -5.6 (-31,26.8) |
| 22 | Southern Sub-Saharan Africa | 0 (0,0) | 10.7 (8.9,12.7) | 55.2 (28.8,88.3) | 0.3 (0.3,0.4) | 0.6 (0.5,0.7) | 26.4 (1.4,51.4) | 10.3 (8.5,11.9) | 15.7 (12.8,18.1) | 26.3 (2.7,52.5) |
